# The Evaluation of a Simulated Interprofessional Education Session Between Dietetics and Acting Students

**DOI:** 10.1111/jhn.70110

**Published:** 2025-08-18

**Authors:** Alison L. Lyles, Marion Scott

**Affiliations:** ^1^ Queen Margaret University Edinburgh UK; ^2^ Renal Dietitian—NHS Lothian Edinburgh UK

**Keywords:** communication, dietetics, experiential learning, interprofessional education, patient‐centred care, simulation

## Abstract

**Introduction:**

This paper evaluates a simulated interprofessional education (IPE) session that brought together dietetic and acting students to explore the development of communication skills and collaborative practice through role‐play consultations. Designed to foster experiential learning, the session enabled dietetics students to practice patient‐centred communication while acting students portrayed patients based on character briefs and offered feedback from a service‐user perspective.

**Methods:**

The session aimed to enhance empathy, rapport‐building, and professional adaptability across both disciplines. Evaluation data were collected via a structured questionnaire completed by 17 dietetic and 5 acting students, assessing areas including skill development, interprofessional collaboration, and session impact.

**Results:**

Results indicated high satisfaction, with 100% of participants rating the session positively and reporting gains in communication, teamwork, and understanding of interdisciplinary roles. Both groups valued the opportunity to apply their respective skills in a realistic, low‐risk setting, highlighting the benefits of including nontraditional disciplines in IPE. Recommendations for future iterations include expanding scenario diversity and providing more time for practice and feedback.

**Conclusion:**

Overall, the session was found to be an effective and engaging method of supporting communication competence and mutual learning between healthcare and performing arts students.

## Summary

- Simulated interprofessional learning activities involving acting and dietetics students can significantly enhance communication, empathy, and teamwork skills relevant to real‐world healthcare practice.
- Role‐play with trained acting students offers a realistic, low‐risk environment for dietetic students to practice and refine patient‐centred consultation skills early in their training.
- Interdisciplinary collaboration between healthcare and performing arts students provides mutual benefits, fostering deeper understanding of professional roles and improving the authenticity of simulated patient interactions.

## 1 Introduction

Interprofessional education (IPE) promotes collaboration among students from different disciplines to prepare them for real‐world healthcare scenarios where teamwork and communication are key [1]. This paper evaluates an IPE session that paired acting and dietetics students to simulate dietetic consultations. The session aimed to develop and improve communication skills and foster a collaborative approach to evaluating communication in healthcare contexts.

Through role‐play and simulated patient interactions, acting students provided a patient portrayal (based on a character‐brief previously provided) while dietetics student practiced conducting consultations with the patient, focusing on rapport‐building and communication strategies. With key outcomes of developing communication strategies and professional perspectives in patient‐centred practice alongside enhancing character development and improvisation, the session also attempted to enable both interprofessional fields to examine empathy and performance within the context of their role.

IPE emphasises that healthcare professionals must collaborate across disciplines to deliver comprehensive patient care [2]. The World Health Organisation (WHO) highlights that IPE enhances communication, respect, and patient outcomes by preparing learners for teamwork [3]. Traditionally, IPE has focused on healthcare fields like nursing, medicine, and allied health, however, expanding these opportunities to include learners from fields like acting and dietetics offers new advantages and perspectives.

Acting students are trained in communication, empathy, and the art of embodying various viewpoints, skills that are essential in high‐quality healthcare interactions. Their curriculum often includes structured training in giving and receiving feedback, reflective practice, and performance critique. Meanwhile, dietetics students are trained to assess nutritional needs, formulate treatment plans including providing accurate and appropriate advice and are required to develop competency in the soft skills of communication, motivational interviewing, empathy and patient‐centred counselling over the course of their practice‐based learning (PBL) training. By pairing these two groups, both cohorts of students are presented with the opportunity to learn from each other through an activity that promotes experiential learning.

Simulation‐based training offers a chance to practice general interaction skills and improve specific aspects of “communication competence”. As an authentic learning method, simulation has the potential to effectively replicate health communication scenarios, allowing for safe experimentation of skill development without the risk of negative consequences [4]. Interprofessional simulations can create a safe environment where healthcare students can rehearse real‐world situations whereby simulations closely mirror the complex, unpredictable nature of real‐life experiences often encountered in healthcare settings [5, 6].

This approach aligns with the growing emphasis on “communication competence” in healthcare education, which emphasises the importance of effective verbal and non‐verbal communication in promoting patient understanding and trust [7]. In this context, we explore the success of an educational collaboration between acting and dietetics students in the potential to provide a valuable opportunity to enable both students to bridge the gap between clinical knowledge and communication skills, and seek to ensure that learners develop not only with technical expertise but also the ability to engage and support patients in a meaningful way.

The simulation of dietetic conversations at an early stage of the PBL journey serves as a platform for dietetic students to begin developing and refining their communication skills. This learning activity, titled “Developing Dietetic Conversations,” was designed with specific objectives for both dietetics and acting students. The dietetics students (typically within their second year of study) aimed to enhance their patient‐centered communication skills whereby they engaged in simulated consultations that allowed them to practice establishing a rapport with service users whilst beginning to navigate more complex communication barriers with professionalism and empathy. Acting students (in the later stages of their program (years 3 & 4) applied their performance techniques to deliver realistic portrayals of patient scenarios by focusing on character development by researching health conditions and traits related to two assigned scenarios. They also provided reflective feedback based on their experience in the role of the service user. The two scenarios that were focused on included: (1) an older adult recently bereaved, experiencing hearing and weight loss, and (2) an adult refugee with type 1 diabetes who has encountered erratic blood sugar levels after recently moving to the UK—this individual speaks English well but may occasionally struggle with certain words.

**Figure 1:**
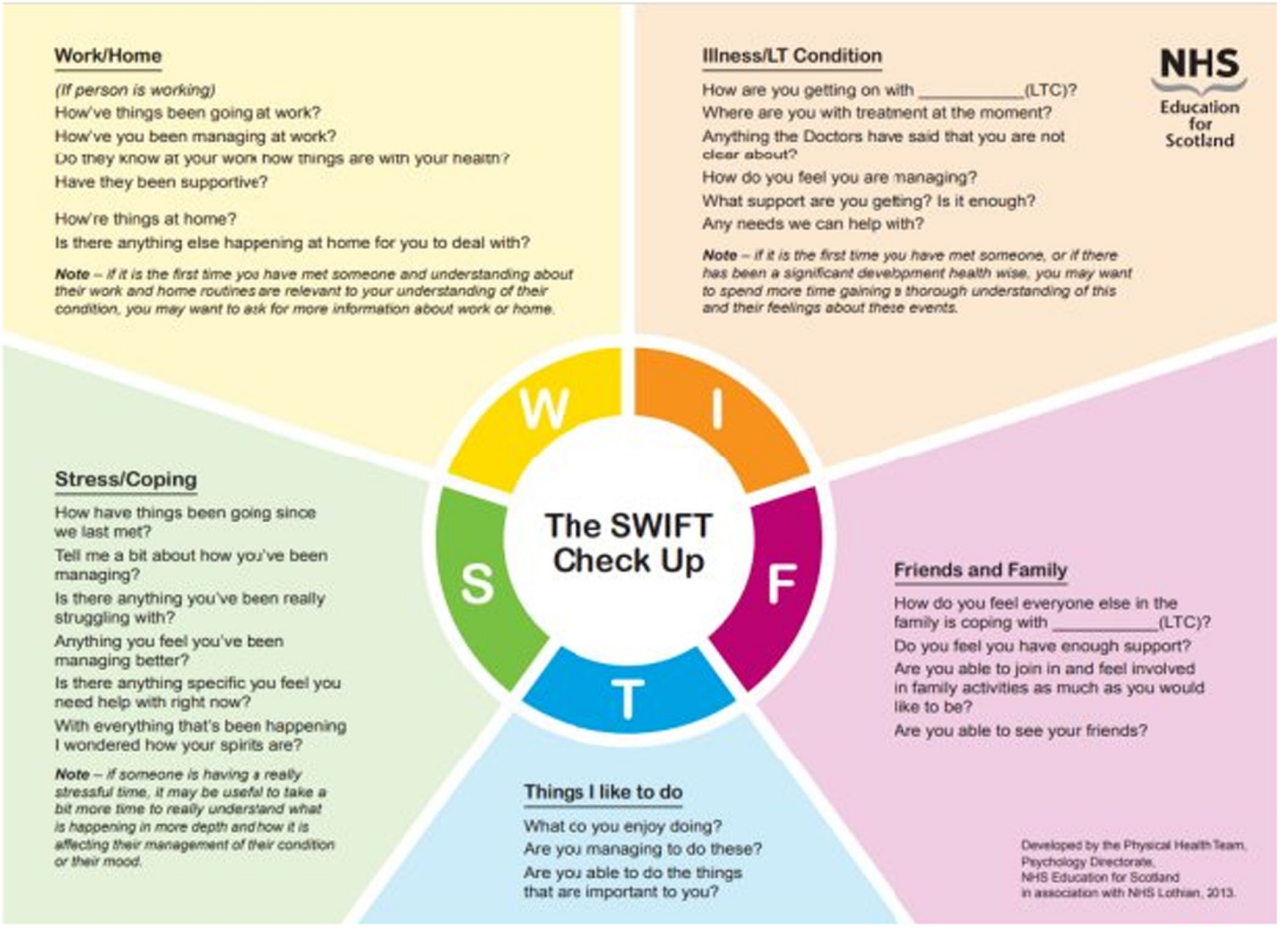
SWIFT Check Up Tool (NHS Education for Scotland).

Before the simulation, dietetic learners participated in a facilitated discussion centred on the patient‐centred communication model, with particular reference to the SWIFT check‐up tool (see Figure 1). As part of this preparatory phase, learners were encouraged to develop patient‐centred questions they could use to guide a conversation with a service user, focusing on the individual's overall health and lifestyle. Students were also asked to identify three potential communication barriers and to consider practical strategies for overcoming these challenges with empathy and professionalism. This simulation represented one of the first structured experiential learning opportunities in the program, particularly for second‐year dietetic students. While some students may have had limited prior exposure to simulation‐based education, this activity was designed as an introductory experience. For acting students—many of whom were in the latter stages of their training—this was one of several simulation experiences incorporated into their curriculum, allowing them to bring depth and realism to their roles while supporting interprofessional learning with other healthcare programmes.

Dietetic students engaged in a 15‐min “dietetic conversation” with acting students, focusing on key communication skills such as rapport‐building, active listening, empathy, professionalism and adapting to communication barriers. Acting students provided feedback through a Consultation and Relational Empathy (CARE) Measure evaluation [8], reflecting upon the consultation from the service user's perspective.

Afterwards, both groups of learners participated in a structured reflective debrief, discussing the successes of their conversations, challenges faced, and areas for improvement.

The paper explores the evaluation of this specific simulated interprofessional learning activity to determine different student perspectives and consider the extent of success of the session in relation to interprofessional collaboration, skill development, practical application, communication and interaction, reflection and impact alongside challenges and suggestions for future improvement.

## 2 Materials and Methods

The learning activity was evaluated through a 21‐point structured questionnaire designed to assess various aspects of the learning experience and to provide insights into the effectiveness of the session relating to skill development, collaboration, and the practical application of theoretical knowledge. The questionnaire was developed collaboratively by members of the teaching team to ensure alignment with the session's intended learning outcomes and underwent internal review for clarity, appropriateness, and readability. While the instrument was straightforward and accessible for student respondents, we acknowledge that future versions would benefit from formal assessment for redundancy and clarity of intent. The questionnaire consisted of a mix of multiple choice and free text questions regarding the following themes: (1) General Evaluation (2) Learning objectives (3) Interprofessional Collaboration (4) Skill Development (5) Practical Application (6) Communication & interaction (7) Session content and delivery (8) Reflection and impact (9) Suggestions for improvement. The specific questions included in the questionnaire can be seen in Table 1.

**Table 1:** Evaluation questionnaire.

#	Question	Response options
**General evaluation**		
1	Overall, how would you rate the effectiveness of this interprofessional education session?	Very good, good, neutral, poor, very poor
2	To what extent did the session meet your learning expectations?	Completely, mostly, moderately, slightly, not at all
**Learning objectives**		
3	Did the session help you understand the role of the other profession (acting/dietetics) in a collaborative healthcare or professional setting?	Completely, mostly, moderately, slightly, not at all
4	To what degree do you feel more confident in collaborating with professionals from other disciplines after this session?	Completely, mostly, moderately, slightly, not at all
**Interprofessional Collaboration**		
5	How well did you work with students from the other discipline (acting/dietetics) during the session?	Very good, good, neutral, poor, very poor
6	Did the session provide opportunities for meaningful collaboration and communication between acting and dietetic students	Completely, mostly, moderately, slightly, not at all
7	How comfortable did you feel working in an interprofessional team with students from a different field?	Very comfortable, comfortable, neutral, uncomfortable, very uncomfortable
**Skills development**		
8	Did you develop any new skills or knowledge that will be useful in future professional practice (e.g., communication, teamwork, understanding different perspectives)?	Yes, no, maybe
9	Were the activities or exercises in the session relevant to your professional role and future career?	Yes, no, maybe
**Practical application**		
10	How well did the session help you apply theoretical knowledge to real‐world scenarios?	Extremely well, somewhat well, neutral, somewhat not well, extremely not well
11	Did you gain insights into how the skills of acting (e.g., empathy, emotional expression) and dietetics (e.g., nutritional education, patient communication) can intersect in professional settings?	Yes, no, maybe
**Communication and interaction**		
12	How effective was the communication between you and students from the other discipline?	Very effective, somewhat effective, neutral, somewhat ineffective, very ineffective
13	Did the session encourage respectful dialogue and the sharing of different perspectives?	Yes, no, maybe
**Session content and delivery**		
14	Was the content of the session clearly explained and easy to understand?	Yes, no, maybe
15	How engaging were the teaching methods and activities used during the session (e.g., role‐play, group discussion, case studies)?	Very engaging, moderately engaging, slightly engaging, not engaging
16	Were the learning materials (e.g., handouts, slides, resources) helpful in supporting your understanding of the session's topics?	Very helpful, moderately helpful, slightly helpful, not helpful
**Reflection and impact**		
17	What is one key takeaway from this interprofessional education session that you will apply in your future career?	Free text
18	How has this session impacted your understanding of interprofessional teamwork in professional practice?	Large impact, moderate impact, small impact, no impact
**Challenges and Suggestions for improvement**		
19	What challenges did you encounter during this session, and how did you overcome them?	Free text
20	What could be improved in future interprofessional education sessions to make them more effective?	Free text
21	Are there any additional topics or activities you would suggest for future interprofessional sessions?	Free text

As the study was undertaken as part of a teaching and learning quality improvement initiative, formal ethical approval was not required. The questionnaire was distributed approximately 4 weeks following the session via Microsoft Forms, and students who participated in the session were invited to respond voluntarily. All responses were collected anonymously.

### 2.1 Data Analysis and Interpretation

Quantitative data from multiple‐choice responses were analysed descriptively, with positive responses summarised as percentages within each student group. Qualitative data from free‐text responses were analysed thematically. Responses were initially reviewed and coded by the main investigator. These codes were then grouped into the predefined thematic categories, with emergent subthemes identified where applicable. We acknowledge that that this would ideally be analysed by two independent reviewers to ensure analytical consistency.

Importantly, data were analysed separately for the two student cohorts involved (acting and dietetic students). This approach reflected best practice in interprofessional education research and allowed for a more nuanced understanding of how different professional groups perceived and engaged with the learning experience.

## 3 Results

From those attending the session, questionnaire responses were collected and analysed. There was a response rate of 17 out of 24 dietetic students and 5 out of 6 acting students.

### 3.1 General Evaluation

Overall, both dietetic and acting students rated the effectiveness of the interprofessional education session highly 100% of students felt the session was either good or very good. The majority of students (88% of dietetic students and 100% of acting students) also felt that the session met their learning expectations either “mostly” or “completely”.

### 3.2 Interprofessional Collaboration

In terms of enhancing students' understanding of the roles of other professionals within a collaborative healthcare environment, both groups reported that the session helped them better understand the role of the other discipline and the majority of students felt more confident in their ability to collaborate with professionals from other fields after the session, with 100% of respondents indicating a moderate to high level of confidence. When evaluating the collaborative aspect of the session, students expressed positive experiences in working with peers from different disciplines. 100% of dietetic and acting students rated their collaboration as either “good” or “very good.” 76% of dietetics students and 70% of acting students felt “comfortable” or “very comfortable” working with students from a different field.

### 3.3 Skill Development

The session contributed significantly to the development of new skills that students deemed relevant to their professional practice—both dietetic and acting students (88% and 100% respectively) were in agreement that they developed new communication and teamwork skills, as well as an enhanced understanding of different professional perspectives. All 100% of respondents agreed that the activities were highly relevant to their future careers.

### 3.4 Practical Application

The session appeared to provide a positive and authentic opportunity for students with 94% of dietetic students and 100% of acting students reporting that they were able to apply the knowledge they had gained to real‐world scenarios. Furthermore, 94% of dietetic students and 100% of acting students recognised how the skills of acting—such as empathy and emotional expression—could intersect with dietetics (e.g., nutritional education, patient communication) in professional settings.

### 3.5 Communication and Interaction

Communication between dietetic and acting students was largely viewed as effective, with 100% of dietetic students and 80% of acting students rating their communication as “somewhat effective” or “very effective.” The session was considered very successful with 100% of students agreeing that the session encouraged respectful dialogue and the sharing of diverse perspectives.

### 3.6 Session Content and Delivery

In terms of session content, 76% of dietetic and 100% of acting students found the material to be clear and easy to understand. The teaching methods, including role‐play and group discussions, were described as very or moderately engaging in 100% of participants.

### 3.7 Reflection and Impact

**Figure 2:**
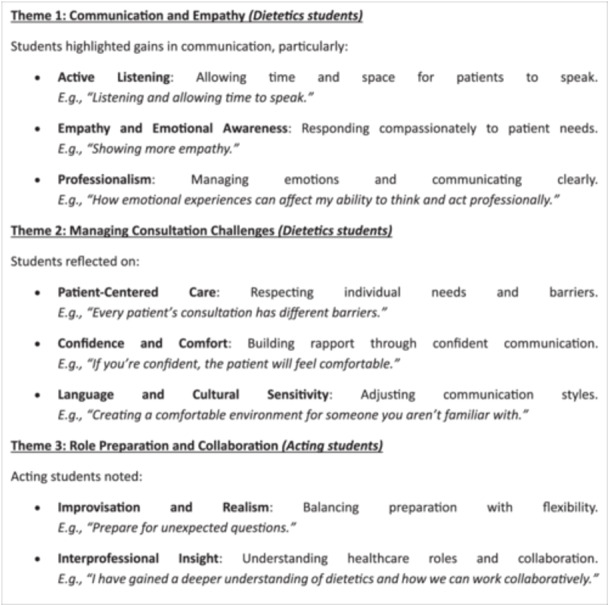
Thematic analysis of responses to the free text question “What is one key takeaway from this interprofessional education session that you will apply in your future career?”.

The impact of the session on students' understanding of interprofessional collaboration was considered significant, with 88% of dietetic and 100% of acting students reporting a “moderate” to “large” impact on their view of teamwork in professional practice in both contexts. From the reflective free text question posed to learners, “What is one key takeaway from this interprofessional education session that you will apply in your future career?”, thematic analysis identified the following three key themes and subthemes relating to communication, empathy, professional preparation and interprofessional collaboration which can be seen in Figure 2 alongside examples of free‐text responses.

### 3.8 Challenges and Suggestions for Improvement

**Figure 3:**
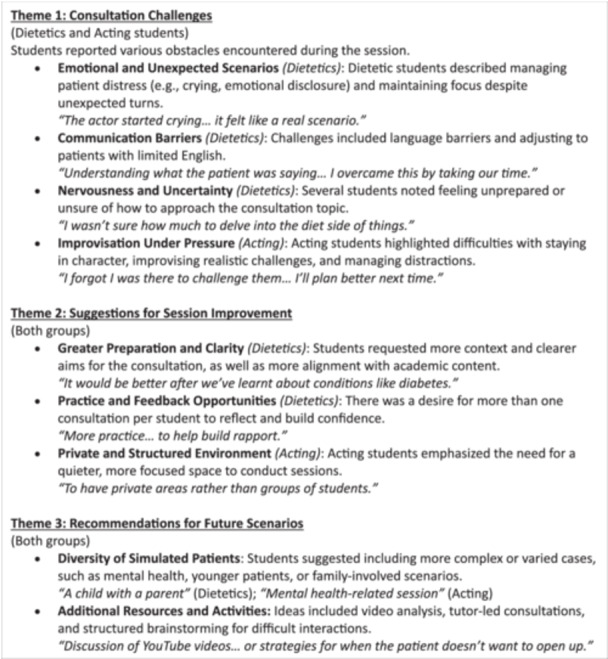
Thematic analysis of responses to questions relating to challenges, improvements and future suggestions.

A total of 32 free‐text responses from dietetic students and 5 from acting students were collected across three free‐text questions relating to challenges, improvements, and future suggestions. Thematic analysis identified several recurring themes and subthemes, which are described in Figure 3. While the session was largely successful, some learners identified challenges, including having a private space to conduct the consultation. Both groups of learners suggested that future sessions could benefit from additional time for additional practice and feedback, as well as more in‐depth consideration of the aim for the consultation. Other suggestions included providing more diverse scenarios that challenge learners to engage with a broader range of interdisciplinary healthcare simulations such as mental health, younger age groups and interaction with carers and additional family members.

## 4 Discussion

The results of the evaluation of this interprofessional education (IPE) session highlight the effectiveness of collaborative learning between dietetic and acting students, outlining its value in the development of essential skills in communication, team working, and professional understanding. Overall, both groups rated the session highly, with the majority of respondents agreeing that it met their learning expectations and provided an excellent introduction to interprofessional collaboration in healthcare through a simulated learning experience.

One of the most notable outcomes from the session was the enhanced understanding of each other's professional roles. Both dietetic and acting students reported that the session helped them better appreciate the contributions of professionals from other disciplines. This aligns with the core objectives of IPE, which aims to break down silos and improve collaboration in healthcare settings [9] and could easily be applied to learners from the wider healthcare sphere. The fact that all students felt more confident in their ability to work with professionals from other fields is particularly promising, as interprofessional collaboration is crucial for improving patient outcomes [10] and developing deeper authenticity in terms of character and role play representations. The positive experience of working in a cross‐disciplinary environment was further evidenced by the high ratings of collaboration, with 100% of both dietetic and acting students describing their interaction as either “good” or “very good”.

Skill development was another key outcome of the session. Both groups reported significant gains in communication and teamwork skills, which they deemed highly relevant to their future careers. The focus on practical application, through role‐play and simulated patient interactions, was central to bridging the gap between theory and practice. The ability to apply theoretical knowledge in real‐world settings is vital in ensuring students are prepared for professional challenges [11]. Notably, both groups acknowledged the value of empathy and emotional expression in enhancing communication with service users. This interprofessional exchange allowed students to understand how seemingly unrelated fields can intersect to improve patient care. Within the session activity debrief, it was clear that some dietetic students valued the actor's depth of emotion and the role of improvisation in creating a sense of realism within the simulation scenario whilst appreciating actor feedback to enable the consideration of “lay‐person” perspectives relating to their ability to adapt their communication strategies. For acting students, there appears to be real benefit to their ability for flexibility and improvisation skills alongside full character immersion, concentration and attention to detail within their character portrayals.

Communication between the two groups was generally rated as effective, particularly by dietetic students, who noted that the session encouraged respectful dialogue and mutual learning. These findings are consistent with the growing recognition of communication competence as a cornerstone of successful interprofessional practice [12] and align with previous conceptual understandings of collaboration between theatre and health [13]. For instance, during the role play, acting students used improvisational techniques to model physical, psychological and emotional barriers to communication, which helped Dietetic students better understand the potential challenging dynamics of clinical interactions. The session not only facilitated the sharing of diverse perspectives but also underscored the importance of ensuring open and respectful communication within healthcare teams and practice.

In terms of content and delivery, the session was well‐received. Most students found the material to be clear and well‐structured, with teaching methods like role‐play and group discussions contributing to a highly engaging learning environment. The satisfaction with the activities suggests that active learning techniques including reflective practice are key to enhancing student engagement.

Despite the overall success, some challenges were identified. Within future sessions there could be clear benefit from incorporating more diverse scenarios that reflect a broader range of healthcare contexts to offer the opportunity for learners to further engage and develop their skills with a wider array of interdisciplinary challenges, enhancing their readiness for real‐world healthcare settings. This presents a potential opportunity to embed a similar simulated activity at a later stage of the learning journey to enhance the development of skills whilst considering more challenging aspects of dietetic consultation such as providing dietary advice and offering dietetic interventions alongside presenting more challenging acting scenarios on which to base character portrayals, perhaps with more pronounced communication and healthcare challenges.

## 5 Conclusion

In conclusion, the evaluation of this interprofessional simulation session highlights its effectiveness in providing a valuable learning experience for both dietetic and acting students, fostering an understanding of interprofessional collaboration and developing key skills that will benefit future professional practice. Certainly, the positive impact on students' views of teamwork and communication reinforces the importance of incorporating IPE into healthcare education but also in widening the symbiotic benefits offered by IPE upon role play and performance for learners in the field of acting.

## Author Contributions

**Alison Lyles:** conceptualisation, methodology, formal analysis, supervision, writing. **Marion Scott:** conceptualisation, resources, formal analysis, writing – review and editing.

## Ethics Statement

The authors have nothing to report.

## Conflicts of Interest

The authors declare no conflicts of interest.

## 1 Peer Review

The peer review history for this article is available at https://www.webofscience.com/api/gateway/wos/peer-review/10.1111/jhn.70110.

## Data Availability

The data that support the findings of this study are available from the corresponding author upon reasonable request.
